# Signaling-mediated control of ubiquitin ligases in endocytosis

**DOI:** 10.1186/1741-7007-10-25

**Published:** 2012-03-15

**Authors:** Simona Polo

**Affiliations:** 1 IFOM, Fondazione Istituto FIRC di Oncologia Molecolare, Via Adamello 16, 20139, Milan, Italy; 2 Dipartimento di Medicina, Chirurgia ed Odontoiatria, Università degli Studi di Milano, Via di Rudinì 8, 20122 Milan, Italy

## Abstract

Ubiquitin-dependent regulation of endocytosis plays an important part in the control of signal transduction, and a critical issue in the understanding of signal transduction therefore relates to regulation of ubiquitination in the endocytic pathway. We discuss here what is known of the mechanisms by which signaling controls the activity of the ubiquitin ligases that specifically recognize the targets of ubiquitination on the endocytic pathway, and suggest alternative mechanisms that deserve experimental investigation.

## 

Ubiquitination modifies proteins in a variety of ways, the significance of which we only partially comprehend. Ubiquitin can be attached: as an individual moiety to a single or multiple lysine residues of substrate (mono- or multiple monoubiquitination); as chains of ubiquitin moieties that are interlinked through any one of the seven lysine residues of ubiquitin (for example K48- or K63-linked chains); or as branched chains, to name but a few [1]. The cell interprets each of these modifications as a distinct signal. The first described role of ubiquitination as mediating protein degradation through targeting to the proteasome has now been complemented with numerous other functions [2]. For example, the signal encoded by K63-linked chains can mediate functions as diverse as receptor endocytosis [3,4], activation of protein kinases in the NF-κB pathway and the initiation of error-free DNA repair [2].

Signal transduction from transmembrane cell surface receptors to nuclear transcription factors is regulated at multiple levels by protein ubiquitination. The covalent attachment of one, or often more, ubiquitin moieties has emerged as the principal mechanism for termination of signaling, by targeting the receptor for endocytosis and, ultimately, degradation in the lysosome [3]. This device controls a vast array of mammalian signaling receptors, such as receptor tyrosine kinases, G-protein coupled receptors (GPCRs), growth hormone receptors, the major histocompatibility complex I, NOTCH, various channels and transporters, and cytokine and interferon receptors [3]. Receptors that are internalized after activation are directed first into the endosomes of the endocytic pathway, and then into multivesicular bodies (MVBs), which undergo a process of maturation that ends with fusion with the lysosome and delivery of the contents for degradation. Ubiquitination of the receptor provides the crucial signal for entering this pathway [3,5-7].

Subsequent delivery of membrane receptors to the lysosome requires accurate recognition of the ubiquitinated cargo by endosomal adaptors and sorting proteins. For the EGF receptor, for example, these are EPS15, epsin and ESCRTs (endosomal sorting complexes required for transport), which contain one or more ubiquitin-binding domains (UBDs) [8]. EPS15 and epsins act at the initial steps of internalization, serving to recruit the enzymes required for ubiquitination of downstream components of the endocytic pathway. ESCRTs act sequentially at various points of the degradative route, sorting the ubiquitinated cargo at the endosomal membrane for inclusion into the intraluminal vesicle of the MVB. ESCRT-0, composed of the two interacting proteins HRS and STAM, is the first in this process. Three additional complexes, ESCRT-I, ESCRTII and ESCRT-III, are then involved in the generation of inward vesicle budding, which is required for MVB maturation (for reviews see [5,9]). The ubiquitin-binding 'route controllers' that operate in this way to ferry the internalized receptor inexorably towards a degradative fate in lysosomes are also inducibly ubiquitinated [10,11] (Figure 1).

**Figure 1:**
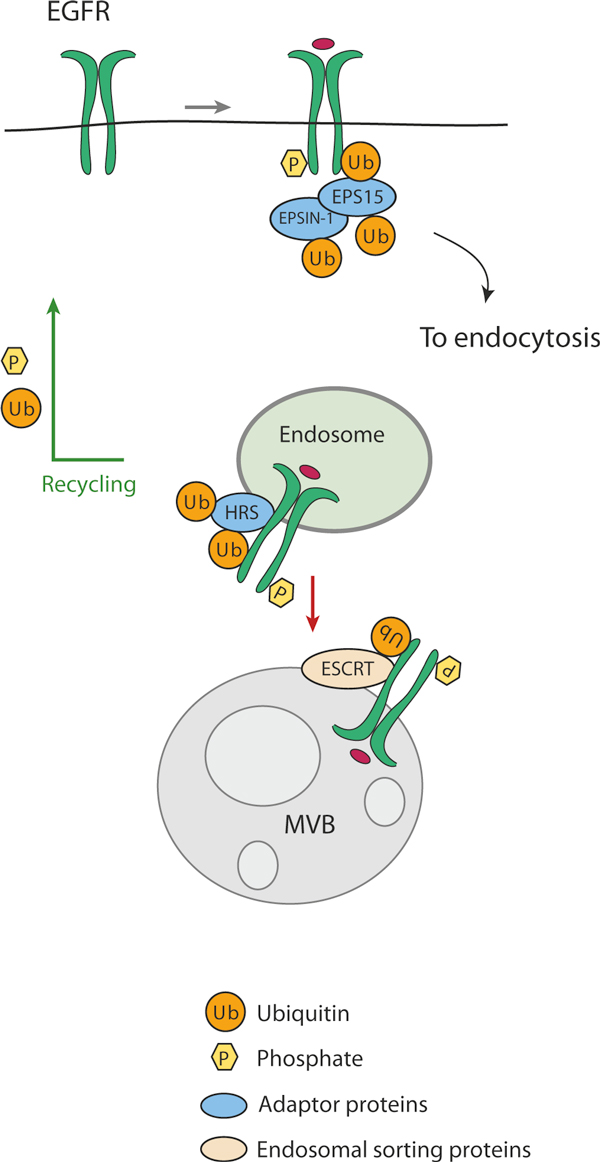
**Receptor internalization and the role of ubiquitin**. The example shown here is that of the EGF receptor. An activated EGFR is ubiquitinated at the plasma membrane and recruits the endocytic adaptor proteins EPS15 and EPSIN-1. These are ubiquitinated, in turn, and direct the internalized receptor to the endosomal pathway where it binds the sorting protein HRS. This is also ubiquitinated and directs progression of the ubiquitinated receptors towards lysosomal degradation through the ESCRT complexes. For simplicity, the EGF receptor is depicted as monoubiquitinated: in reality, it is both multimono- and polyubiquitinated. MVB, multivesicular body.

The inducibility of the system illustrates the dynamic nature of ubiquitin-based endocytic regulation. Indeed, over the past 15 years, studies from different laboratories have revealed a critical role for ubiquitination in receptor down-regulation, a process that is essential for the spatial and temporal resolution of receptor signaling [3].

The central component of this regulatory pathway is the E3 ligase that attaches the ubiquitin molecules to the receptor, or to the ubiquitin-binding endocytic adaptors (Figure 1). A hierarchical set of three types of enzyme is required for substrate ubiquitination: ubiquitin-activating (E1), ubiquitin-conjugating (E2) and ubiquitin-protein ligase (E3) enzymes. In mammals two E1-activating enzyme transfer ubiquitin to roughly three dozen E2s, which function together with several hundred different E3 ubiquitin ligases to ubiquitinate thousands of substrates. In the endocytic pathway distinct E3 enzymes generally catalyze the ubiquitination of the cell-surface receptors and the endosomal sorting proteins [3]. Therefore the endocytic sorting of a given target generally involves more than one E3 ubiquitin protein ligase.

Here we focus on how signaling controls the activity of the E3 ligases that ubiquitinate receptors and endocytic adaptors. Although E3 ligases have largely been considered to be constitutively active and regulated only at the level of target binding, it has recently become evident that they are subject to regulation by either E3 or substrate phosphorylation, or by exploitation of adaptor proteins that facilitate E3 activity. We discuss here the various ways of regulating E3 ligases in the context of endocytosis.

## Ligand-induced E3 ligases for receptor ubiquitination

**Figure 2:**
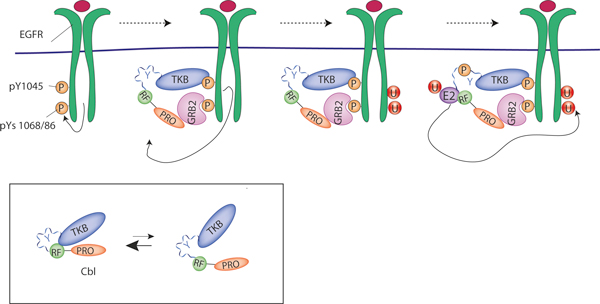
**EGFR ubiquitination by Cbl**. Upon EGF-dependent receptor activation, the GRB2-Cbl complex binds to the receptor through interactions of: i) the SH2 domain of GRB2 with pY1045 of EGFR, and ii) the TKB domain of Cbl (either c-Cbl or Cbl-b) with pY1068 or pY1086. This substrate interaction may either stabilize or select for a partially open Cbl conformation (see bottom panel and main text). EGFR-bound Cbl becomes phosphorylated on a critical tyrosine, leading to full rotation of the linker region. This, in turn, exposes the RING domain for ubiquitin-charged E2 binding, resulting in the allosteric activation of the E2 by Cbl and ubiquitination of the EGFR. Note that, to simplify the picture, Cbl bound to one receptor molecule is depicted to ubiquitinate the other molecule of the dimer. No available data suggest that this is indeed the case. For simplicity, the EGF receptor is depicted as monoubiquitinated: in reality, it is both multimono- and polyubiquitinated.

Protein phosphorylation, which is well known to function in recruitment of the ubiquitination machinery to the substrate [12], may also act directly to regulate the activity of distinct ubiquitin ligases. The best-characterized circuitry involves the E3 ligase Cbl, which is responsible for the ubiquitination of several receptor tyrosine kinases (RTKs) [3]. The mammalian Cbl protein family consists of the three homologs c-Cbl, Cbl-b, and Cbl-3, all of which associate with a wide variety of signaling proteins [13]. Two highly conserved amino-terminal domains contribute strongly to E3 regulatory function. First, the amino-terminal tyrosine kinase binding (TKB) domain of Cbl recognizes phosphotyrosine residues and allows Cbl to interact directly with activated RTKs at the plasma membrane (Figure 2). Second, the RING finger domain recruits ubiquitin-loaded E2s, whose interaction with Cbl results in the ubiquitination and subsequent degradation of the associated RTK. In the case of the epidermal growth factor receptor (EGFR) and the hepatocyte growth factor receptor MET, the molecular mechanism of receptor ubiquitination has been investigated in detail. In both cases, Cbl binds directly to phosphotyrosine (pY)-sites on the activated receptor through its TKB [14,15], as well as indirectly through its constitutive partner GRB2, which is recruited to receptors via other pY sites [7,16-18]. Both direct and indirect interactions of Cbl with the EGFR or MET are required for full ubiquitination of these receptors (Figure 2). Once bound, the ligase is phosphorylated and consequently activated [19]. Two structural studies have now shed light on the mechanism of phosphorylation-induced activation of c-Cbl and Cbl-b [20,21]. In the absence of substrate binding, the TKB and RING domains form a compact structure that masks the E2 binding site. Binding of the TKB to the substrate induces a first rotation of the linker region, allowing phosphorylation of tyrosine 371 (363 in Cbl-b). This phosphorylation event induces a complete rotation of the linker region that unmasks the RING E2 binding surface and activates the ligase [20,21].

Another class of E3 ligases, the HECT NEDD4 family [22], whose regulation has been extensively studied, also regulates endocytosis and sorting of numerous signaling receptors [3,5-7]. These enzymes present a conserved modular organization with an amino-terminal C2 domain that is crucial for membrane localization, between two and four WW domains capable of recognizing substrates and adaptor proteins through PY motifs, and a carboxy-terminal catalytic HECT domain. In contrast to RING-based ligases in which the RING is an allosteric activator of the E2, HECT-containing E3s have intrinsic catalytic activity and directly ubiquitinate their targets. In humans, there are nine members of this family: NEDD4 (also known as NEDD4-1), NEDD4L (also known as NEDD4-2), ITCH (also known as AIP4), WWP1, WWP2, SMURF1, SMURF2, NEDL1 (also known as HECW1) and NEDL2 (also known as HECW2). Rsp5 is the unique, essential member of the NEDD4 family in *Saccharomyces cerevisiae*. In normal conditions most of them appear to be in an inactive state because of an intramolecular inhibitory interaction between the carboxy-terminal HECT and the amino-terminal C2 domain (in the case of SMURF2, NEDD4 and WWP2 [23]) or the WW domains (in the case of ITCH [24]). Activation of this class of enzyme can occur in various ways that are briefly described below.

**Figure 3:**
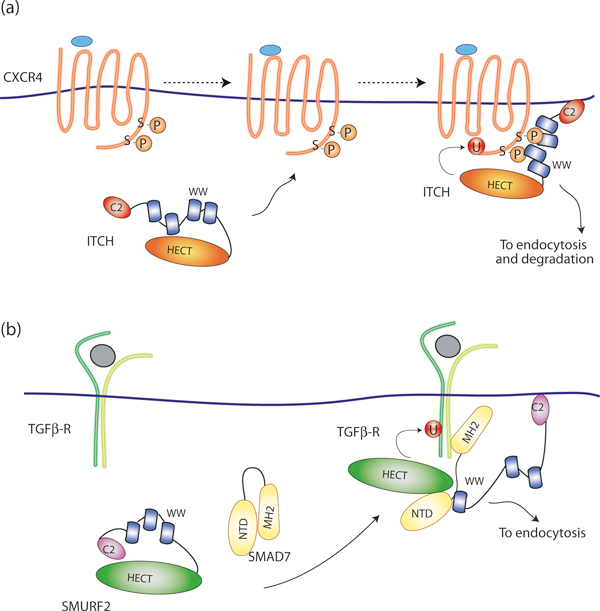
**Activation of E3 ubiquitin ligases through recruitment to activated receptors**. **(a) **Ubiquitination of CXCR4 by ITCH. ITCH activity is inhibited as a result of the intramolecular interaction between the WW domain and the carboxy-terminal catalytic HECT domain. Upon agonist-mediated activation, CXCR4 becomes phosphorylated at Ser324 and Ser325 by an unknown kinase. This leads to the recruitment of the ITCH, through its WW domain, and consequent release of the inhibitory intramolecular interaction, allowing ubiquitination of the receptor. **(b) **Ubiquitination of the TGF-β receptor by the SMURF2-SMAD7 complex. SMURF2 activity is inhibited as a result of the intramolecular interaction between the amino-terminal C2 and the carboxy-terminal catalytic HECT domain. The interaction with SMAD7 NTD displaces the C2 domain of SMURF2 from the HECT domain and activates the ligase. The activated SMURF2-SMAD7 complex associates with activated TGF-β receptor complexes at the membrane via the displaced C2 domain, causing receptor ubiquitination.

ITCH is the E3 ligase for the chemokine receptor CXCR4 [25]. The ubiquitin moiety on CXCR4 serves as a signal on endosomes for entry into the degradative pathway and long-term attenuation or downregulation of signaling [25]. ITCH can interact directly with CXCR4 through a non-canonical WW domain-mediated interaction involving serine residues within the carboxy-terminal tail of CXCR4. These serine residues are phosphorylated upon agonist activation, and are critical for mediating agonist-promoted binding of ITCH and the subsequent ubiquitination and degradation of CXCR4 [26] (Figure 3a). Also in this case, the ligase appears to be regulated by phosphorylation. ITCH phosphorylation is activated by JNK1 [24], and is thought to lead to conformational changes that disrupt the inhibitory intramolecular interactions between its WW and the HECT domains.

In the case of SMURF2, autoinhibition of the HECT domain by the C2 domain helps in maintaining the steady-state levels of this E3 ligase and can be relieved by adaptor-mediated substrate targeting [23]. SMURF1 and SMURF2 bind to TGF-β family receptors through the inhibitory Smad proteins, SMAD6 and SMAD7, to induce their ubiquitin-dependent degradation. Wiesner *et al. *[23] demonstrated that intramolecular interactions between the C2 and HECT inhibit SMURF2 catalytic activity interfering with ubiquitin thioester formation. This *in cis *autoinhibition can be relieved by binding of the amino-terminal domain (NTD) of the adaptor protein SMAD7 to the E3 HECT domain (Figure 3b). The SMAD7 NTD further enhances the catalytic activity of the SMURF2 ligase by recruiting the E2 UbcH7 to the HECT domain [27]. By releasing C2-mediated autoinhibition, stimulating E2 binding, and recruiting SMURF targets, SMAD7 functions at multiple levels to control E3 activity and ensure specificity in SMURF-catalyzed ubiquitination.

Recently, a role for a UBD present on the N-lobe of the HECT domain of NEDD4 and Rsp5 has been identified [28,29]. The ability of the HECT domain to bind non-covalently to the distal ubiquitin at the growing end of the polyubiquitin chain on the substrate allows enzyme processivity [28]. It is tempting to attribute an inhibitory role of the C2 binding for this critical feature of these enzymes. Accessibility of the UBD may be restored in response to upstream signaling events capable of inducing phosphorylation and/or ubiquitination of critical sites in the C2 or in the HECT domain, leading to full ligase activation. While this hypothesis needs to be experimentally verified, we notice that ubiquitination of NEDD4 is a critical event for the coupled monoubiquitination of EPS15 ([30] and below).

**Figure 4:**
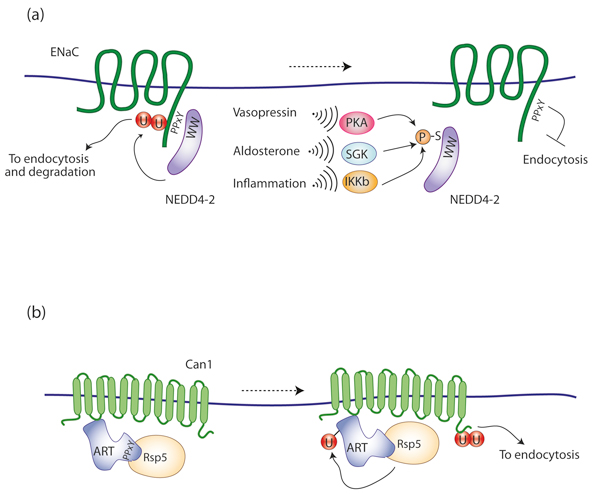
**Regulation of channels and transporters by ubiquitination. (a) **ENaC ubiquitination by NEDD4-2. NEDD4-2 binds to PY motifs on the epithelial Na^+ ^channel ENaC and catalyzes its ubiquitination. This induces ENaC endocytosis and lysosomal targeting, resulting in fewer channels at the cell surface. To increase Na^+ ^transport, NEDD4-2 is phosphorylated by kinases, including PKA, SGK, and IKKβ, in turn activated by various signaling pathways. Phosphorylation of NEDD4-2 induces binding of 14-3-3 dimers (not shown), which prevents NEDD4-2 from binding to ENaC. As a result, endocytosis of ENaC is inhibited, and increased ENaC presence at the surface enhances epithelial Na^+ ^absorption. **(b) **Rsp5 ubiquitinates permeases and transporters. In yeast, arrestin-related endocytic adaptors (ARTs) and the E3 ubiquitin ligase Rsp5 are recruited to the plasma membrane in response to environmental stimuli that trigger the endocytosis of proteins such as permeases and transporters (for example, the arginine transporter Can1). Through their PY motifs, ARTs bind to the WW domain of Rsp5 and mediate ubiquitination of cargo. ARTs are also ubiquitinated by Rsp5, an event required for endocytosis.

In some cases, such as for the epithelial Na^+ ^channel (ENaC), receptor:ligase interaction - and consequent receptor ubiquitination - is the default pathway, with phosphorylation negatively regulating ligase activity. NEDD4-2 binds constitutively to ENaC PPxY-containing motifs and catalyzes its ubiquitination, internalization and lysosomal targeting. This prevents Na^+ ^overload in epithelial cells and is necessary for the maintenance of salt and fluid balance in the body. To increase ENaC abundance at the surface and enhance epithelial Na^+ ^absorption, NEDD4-2 is phosphorylated by various kinases, including protein kinase A (PKA), serum- and glucocorticoid-inducible kinase (SGK), and IκB kinase (IKK)β (Figure 4a). Phosphorylation induces binding of 14-3-3 proteins, which prevents NEDD4-2 from binding to ENaC [31-33].

## Regulation of receptor ubiquitination by adaptor proteins

Finally, specific binding proteins can regulate the process of ubiquitination by acting as adaptors to recruit E3 proteins to receptors that lack a direct binding motif for the ligase (reviewed in [34]). The best evidence for this mechanism so far comes from membrane transport directed by the yeast HECT E3 ligase Rsp5, which is the unique homolog of the mammalian NEDD4 family proteins. While in humans there are nine members of the NEDD4 family, yeast Rsp5 is sufficient on its own to control most membrane traffic ubiquitination events at the plasma membrane and at other biomembranes [35]. Cooperation with adaptors such as Bul1/Bul2, Bsd2/Tre1/Tre2, or Ear1/Ssh4 enables Rsp5 to cope with this large number of substrates [35], and the discovery of the yeast family of ARR-related proteins (ARTs), which direct Rsp5 activity to various plasma membrane receptors [36-38], shows that the adaptor mechanism is even more extensive than previously thought (Figure 4b). Does receptor signaling regulate these HECT adaptor proteins? Two recent papers provide evidence that it does [39,40]. MacGurn *et al. *[40] demonstrated that signaling from TORC1, which is a central regulator of cell growth in response to amino acid availability, regulates Rsp5 targeting and endocytosis of nutrient transporters at the plasma membrane. The effector mechanism that regulates endocytosis involves a TORC1-Npr1 negative kinase signaling cascade that tunes the phosphoinhibition of the ubiquitin ligase adaptor Art1 [40]. Leon and colleagues [39] identified the α-arrestin Rod1/Art4 as a direct target of the glucose signaling pathway. Glucose promotes Rod1/Art4 dephosphorylation and its subsequent release from a phospho-dependent interaction with 14-3-3 proteins. This allows Rsp5-mediated Rod1 ubiquitination, a prerequisite for transporter endocytosis [39]. It is conceivable that other signaling pathways may similarly regulate the activity of other ART family proteins.

Does this mechanism also operate in mammals? And if yes, how? These important gaps in our understanding need to be filled. Of note, arrestin domain containing protein 3 (ARRDC3) was recently shown to interact with NEDD4 and to be an essential adaptor for β2-adrenergic receptor (β2AR) ubiquitination [41].

## Ligand-induced E3 ligases for adaptor ubiquitination

**Figure 5:**
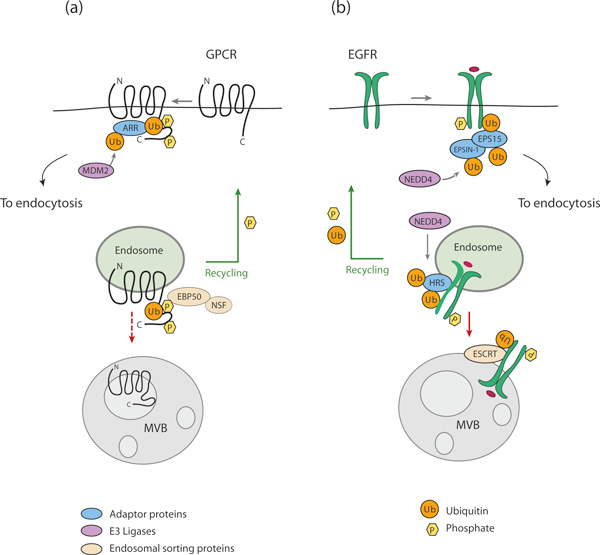
**Ubiquitination of adaptors**. **(a) **Agonist induces rapid ubiquitination of GPCR-recruited ARR by MDM2, a process required for receptor internalization. Once internalized, GPCRs can be dephosphorylated and rapidly recycled to the plasma membrane through a mechanism that involves the sorting proteins EBP50 and NSF. **(b) **Activated EGFR is ubiquitinated at the plasma membrane by Cbl and recruits the UBD-containing endocytic adaptors EPS15 and EPSIN-1 at the plasma membrane, and subsequently HRS at the endosomal membrane. These adaptors, in turn, are ubiquitinated by NEDD4 through a process known as coupled monoubiquitination. This directs progression of the ubiquitinated receptors toward lysosomal degradation through the ESCRT complexes. A similar mechanism can be envisioned for other RTKs.

The adaptors we have just discussed function to recruit E3 ligases to receptors. We now return to the endocytic adaptors that are recruited to the ubiquitinated receptors and direct their subsequent endocytosis. As with direct receptor ubiquitination, the ubiquitination of endocytic adaptors plays a critical role in endocytosis. The arrestin (ARR) family of proteins is able to direct internalization of GPCR cargo. Signaling from activated GPCRs is terminated when GPCRs are phosphorylated by GPCR kinases (GRKs), leading to the recruitment of ARR, which binds to AP-2 and clathrin, structural components of the vesicles formed at the plasma membrane causing the whole complex to be internalized. Agonist-stimulated ubiquitination of ARR mediated by the E3 ligase murine double minute (MDM2), an important negative regulator of p53, is critical for rapid receptor internalization [42]. MDM2-ARR binding is constitutive and does not persist after receptor activation, suggesting that ubiquitin modification might cause a conformational change on ARR required to promote internalization. GPCRs themselves can also be ubiquitinated, most probably by NEDD4, an event required for cargo degradation but not internalization [43]. Thus by analogy with the 'phosphorylation code' on the receptor carboxy tail, ubiquitin modifications on both adaptors and receptors result in a 'ubiquitination code' that fine-tunes signal strength, localization, and cellular functions of GPCR (Figure 5a).

ARR is not the sole example of an endocytic adaptor subjected to ubiquitin modification. Several components of the downstream endocytic machinery are monoubiquitinated upon RTK activation [10,11,44,45]. In most cases, these adaptors are ubiquitin receptors that are ubiquitinated by the E3 ligase NEDD4. The presence of a UBD is required for monoubiquitination of the UBD-harboring adaptor, in a process termed coupled monoubiquitination whose molecular workings have been elucidated using the endocytic proteins EPS15 and EPSIN-1 as models [30,46] (Figure 5b). By contrast, the mechanism by which NEDD4 recruitment is induced by the activated EGFR remains to be clarified.

What is the role of adaptor ubiquitination? Monoubiquitination might permit the formation of several tiers of ubiquitination-dependent interactions in the endosome, by allowing binding of ubiquitinated cargo (through UBDs) and recruiting another layer of ubiquitin receptors through the monoubiquitin signal. The result would be signal amplification and progression of ubiquitinated cargoes along the endocytic pathway.

Monoubiquitination of ubiquitin receptors may also result in an intra-molecular interaction between their UBDs and monoubiquitinated residues, with resulting dissociation from the ubiquitinated cargo [47,48]. These two possibilities are not mutually exclusive and both mechanisms may be involved in the regulation of endocytic processes, possibly by acting at distinct steps and/or regulating different endocytic adaptors.

## Is the ubiquitination cascade like the phosphorylation cascade?

It is important to realize that the power of ubiquitin stems from its capacity to act as a protein-protein interaction module that targets substrates to a plethora of downstream effectors. In order to realize this network, complex molecular machines generate and recognize signal diversity based on ubiquitin-binding modules. The network is fine-tuned by ubiquitinating (E3 ligases) and deubiquitinating enzymes (DUBs) that balance the absolute levels of protein ubiquitination, as well as the abundance and localization of adaptors that contain docking sites for specific ubiquitinated proteins (UBDs) [49].

One such network pivots around EGFR. EGF stimulation promotes ubiquitination of EGFR and of EGFR endocytic adapters, providing a striking example of concerted regulation of signaling, ultimately regulating the route of EGFR internalization [50,51]. Our own approach to understanding the complex interplay between EGFR-induced signaling circuitries has been the recent elucidation of the EGF-induced 'ubiproteome' [52]. This work has uncovered an extensive ubiquitin-based signaling network that impinges on a wide array of signaling circuitries and various aspects of cellular physiology including DNA repair, nuclear transport, mRNA processing, metabolic pathways, and ribosome biogenesis. Interestingly, many ubiquitin machinery enzymes were detected in the EGF-induced ubiproteome. Regardless of the initial triggering mechanism (which necessarily involves the kinase activity of the EGFR), the ubiquitin signal seems to be rapidly transmitted to, and amplified through, the ubiquitin machinery. Just as in the phosphorylation cascade, in which critical enzymes such as kinases and phosphatases are often activated by phosphorylation, ubiquitinating enzymes appear to be regulated by ubiquitination. Moreover, a comparison of the EGF-induced ubi- and pY-proteomes revealed a significant overlap and identified many highly connected 'hub' proteins that are both phosphorylated and ubiquitinated [52]. These data suggest that two complementary and interlinked enzymatic cascades drive the flow of information from receptors to downstream signaling molecules: kinases/phosphatases and E3 ligases/DUBs. In essence, these two post-translational-modification-based networks can be conceptualized as two overlapping, diffusely interconnected, matrices through which external signals are transduced and interpreted by the cell. To understand how this is achieved, a multidisciplinary approach is required. No single 'omics' analysis can fully unravel the complexities of the system. Complete understanding will be achieved only by integrating information from high-resolution molecular investigations, 'omics' approaches and 'top-down' systems-based modeling.
